# Identification of Novel Imidazo[1,2-*a*]pyridine Inhibitors Targeting *M. tuberculosis* QcrB

**DOI:** 10.1371/journal.pone.0052951

**Published:** 2012-12-31

**Authors:** Katherine A. Abrahams, Jonathan A. G. Cox, Vickey L. Spivey, Nicholas J. Loman, Mark J. Pallen, Chrystala Constantinidou, Raquel Fernández, Carlos Alemparte, Modesto J. Remuiñán, David Barros, Lluis Ballell, Gurdyal S. Besra

**Affiliations:** 1 School of Biosciences, University of Birmingham, Edgbaston, Birmingham, United Kingdom; 2 Diseases of the Developing World, GlaxoSmithKline, Tres Cantos, Madrid, Spain; Université de Montpellier 2, France

## Abstract

*Mycobacterium tuberculosis* is a major human pathogen and the causative agent for the pulmonary disease, tuberculosis (TB). Current treatment programs to combat TB are under threat due to the emergence of multi-drug and extensively-drug resistant TB. Through the use of high throughput whole cell screening of an extensive compound library a number of imidazo[1,2-*a*]pyridine (IP) compounds were obtained as potent lead molecules active against *M. tuberculosis* and *Mycobacterium bovis* BCG. The IP inhibitors (**1**–**4**) demonstrated minimum inhibitory concentrations (MICs) in the range of 0.03 to 5 µM against a panel of *M. tuberculosis* strains. *M. bovis* BCG spontaneous resistant mutants were generated against IP **1**, **3**, and **4** at 5× MIC and subsequent whole genome sequencing identified a single nucleotide polymorphism ^937^ACC>^937^GCC (T313A) in *qcrB*, which encodes the b subunit of the electron transport ubiquinol cytochrome C reductase. This mutation also conferred cross-resistance against IP **1**, **3** and **4** demonstrating a common target. Gene dosage experiments confirmed *M. bovis* BCG QcrB as the target where over-expression in *M. bovis* BCG led to an increase in MIC from 0.5 to >8 µM for IP **3**. An acute murine model of TB infection established bacteriostatic activity of the IP series, which await further detailed characterization.

## Introduction

The Gram-positive bacillus, *Mycobacterium tuberculosis,* is a major human pathogen and is the causative agent of tuberculosis (TB). This infectious disease poses a global health risk with an incidence rate of 8.8 million cases and a fatality rate of 1.4 million [1], [2], [3]. Co-infection with Human Immunodeficiency Virus (HIV) augments the number of TB cases and the development of active tuberculosis [1], [3]. As such, there remains an urgent requirement for new anti-tubercular drugs [4]. This has been further compounded by the emergence of drug resistance that has rendered existing treatment programs ineffective. In 2010, an estimated 650,000 cases of multi-drug resistant TB (MDR-TB) were reported [5] and since then, extensively-drug resistant TB (XDR-TB) and totally-drug resistant TB (TDR-TB) have been established [6]. Evidently, the development of successful anti-tubercular agents is imperative, but simultaneously faces a myriad of challenges. These include: meeting the directives of shortening treatment duration; dosing frequency; co-administration with HIV medications; reducing adverse effects [4]. Thus, to circumvent an era where TB is untreatable, the discovery of unique drug targets and novel inhibitory compounds can be considered invaluable in terms of meeting the current and future therapeutic needs to relieve the burden of TB cases worldwide [4].

Tackling this problem, many researchers in the area of drug discovery are now shifting from single-enzyme to whole cell phenotypic approaches, using High Throughput Screening (HTS) of extensive compound libraries [7], [8], [9], [10]. For example, the diarylquinoline family of *M. tuberculosis* inhibitors were identified utilizing a whole cell phenotypic HTS campaign of a library of more than 70,000 compounds against *Mycobacterium smegmatis* [11], [12], [13]. The lead compound diarylquinoline TMC207 was then subsequently identified as a potent inhibitor of the *M. tuberculosis* ATP synthase through whole genome sequencing of spontaneous resistant mutants [11], [12]. In addition to the potency of TMC207 against both drug-sensitive and MDR-TB strains, the recent success in Phase II clinical trials places TMC207 as a future front-line anti-tubercular agent [13]. Similarly, the *M. tuberculosis* inhibitors SQ109 [14], [15], [16] adamantly ureas [17], [18], and benzimidazole [19] were identified following HTS campaigns and chemical lead optimization. The cellular target of SQ109 [20], adamantly ureas [17], pyrrole BM212 [21], and benzimidazoles [19], has recently been identified by whole genome sequencing of spontaneous resistant mutants generated against each inhibitor series, which revealed the common target MmpL3, a membrane transporter involved in the export of trehalose monomycolate (TMM) and cell wall biosynthesis [17], [20], [21], [22].

**Figure 1:**
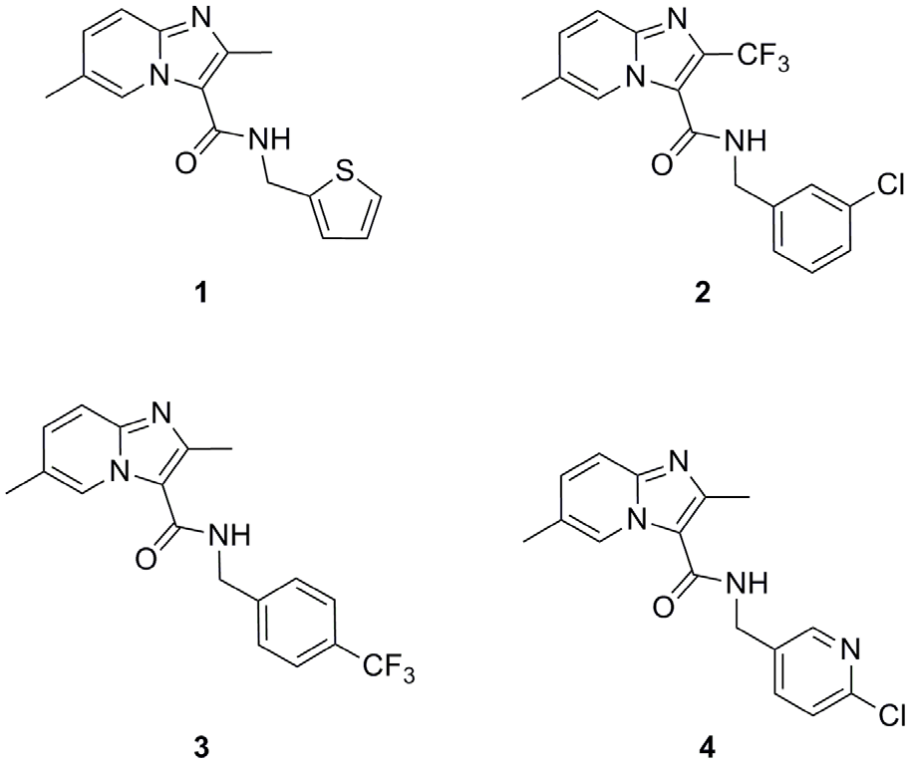
Compounds from the IP series active against *M. tuberculosis*. Structures of the initial IP hits (**1** and **2**) identified in the HTS campaign against *M. bovis* BCG (activity later confirmed in *M. tuberculosis*) and of optimized compounds **3** and **4**.

Another inhibitor series found to have anti-TB activity are the imidazo[1,2-*a*]pyridine-3-nitroso compounds, but they exhibit undesirable toxicity in a VERO cell line [23]. A similar family of compounds, the imidazo[1,2-*a*]pyridine-3-hydrazones, have been synthesized but are all inactive against *M. tuberculosis* H37Rv [24]. More recently, 3-amino-imidazo[1,2-*a*]pyridines were shown as *M. tuberculosis* glutamine synthetase inhibitors [25]. The anti-TB properties of the 2,7-dimethylimidazo[1,2-*a*]pyridine-3-carboxamides have also been investigated [26]. These compounds are synthetically tractable, possess druggable properties, and have excellent selective potency against MDR- and XDR-TB [10], [27]. We have performed a HTS campaign, which has also identified imidazo[1,2-*a*]pyridines (IP) as potent inhibitors of *M. tuberculosis* and *M. bovis* BCG. Herein we describe four inhibitors of the IP series (Figure 1, Table 1) and demonstrate that IP specifically targets QcrB, which encodes the b subunit of the electron transport ubiquinol cytochrome C reductase.

**Table 1:** Preliminary anti-tubercular activity, cytotoxicity and microsomal stability profile of the HTS hits identified in the IP series.

	MIC (µM)						Tox50 (µM)	Mouse		Human	
IP	H37Rv^b^	Beijing^b^	inh233^b^	CDC1551^b^	*M. bovis* BCG	Anti-bacterial panel	Cell lines^a^	CLint(ml/min/gprotein)	T1/2(min)	CLint(ml/min/gprotein)	T1/2 (min)
**1**	0.2	0.03	0.03	0.06	1	all>64	all>25	>30	<3	3.9	21
**2**	0.2	0.06	0.03	0.06	ND	all>16	all>50	>30	<3	>30	<3
**3**	0.03	ND^c^	ND	ND	0.5	all>16	all>25	7	11.6	4.9	15.8
**4**	5	ND	ND	ND	5	all>16	all>50	7.4	11.4	2.3	>30

A small representative set of Gram-positive and Gram-negative organisms were analyzed in addition to the *mycobacteria*: *Enterococcus faecium*, *Enterococcus faecalis*, *Haemophilus influenzae*, *Moraxella catarrhalis*, *Streptococcus pneumoniae*, *Escherichia coli* and *Streptococcus pyogenes*.

a L1210, HepG2, NEURO 2A, MDCK and H9C2(2-1).

b *M. tuberculosis* strains.

c ND, Not determined.

## Materials and Methods

### Ethics Statement

All experiments were approved by the Diseases of the Developing World (DDW-GSK) ethical committee. The animal research complies with Spanish and European Union legislation (European directive 86/609/EEC) on animal research and GlaxoSmithKline 3R policy on the care and use of animals: Replacement, Reduction and Refinement.

### General Information

All commercially available reagents and solvents were used without further purification. Automated flash chromatography was performed on a Biotage FlashMaster II system with peak detection at 254 nm. All products were obtained as amorphous solids and melting points were not measured. ^1^H NMR spectra were recorded at 300 MHz on a Varian spectrometer. Chemical shifts (δ) are given in ppm relative to the solvent reference as an internal standard (d^6^-DMSO, δ = 2.50 ppm). Data are reported as follows: chemical shift (multiplicity (s for singlet, d for doublet, t for triplet, m for multiplet, br for broad), integration, coupling constant(s) in Hz). HPLC–MS analyses were conducted on an Agilent 1100 instrument equipped with a Sunfire C_18_ column (30 mm x 2.1 mm i.d., 3.5 mm packing diameter) at 40°C coupled with a Waters ZMD2000 mass spectrometer; the method of ionization was alternate-scan positive and negative electrospray. Compounds had purity of >98%, as determined by HPLC and ^1^H NMR analysis. All commercially available compounds, including hit molecules **1** and **2,** were used without further purification.

### Chemical Synthesis of Compounds 3 and 4

2,6-Dimethyl-N-(4-(trifluoromethyl)benzyl)imidazo[1,2-*a*]pyridine-3-carboxamide (**3**). To a mixture of 2,6-dimethylimidazo[1,2-*a*]pyridine-3-carboxylic acid (2.5 g, 13.14 mmol) and dichloromethane (200 ml), N-(3-dimethylaminopropyl)-N′-ethylcarbodiimide hydrochloride (3.78 g, 19.72 mmol), 1-hydroxy-1H-benzotriazol hydrate (3.02 g, 19.72 mmol) and N,N-diisopropylethylamine (2.68 ml, 15.77 mmol) were added. After stirring for 15 minutes at room temperature, (4-(trifluoromethyl)phenyl)methanamine (1.873 ml, 13.14 mmol) was added. The reaction was stirred at room temperature for 4.5 hours and NaHCO_3_ (200 ml) was added. The organic layer was washed with NaCl (200 ml) and dried over Na_2_SO_4_, filtered, and concentrated under reduced pressure. The crude mixture was purified using a silica gel cartridge (Analogix) and eluted with a mixture of methanol/dichloromethane (gradient 0–5%). Collected fractions were evaporated under reduced pressure to obtain the title compound **3** (4.0 g, 11.52 mmol, 88% yield) as a white solid. MS (m/z) = 348 ([M+H]^+^); ^1^H NMR: δ 8.86 (br s, 1H), 8.37 (br t, 1H, *J* = 5.8), 7.72 and 7.60 (AA′BB′ system, 4H), 7.49 (d, 1H, *J* = 9.1), 7.26 (dd, 1H, *J* = 9.1 and 1.8), 4.61 (d, 2H, *J* = 6.1), 2.60 (s, 3H), 2.30 (s, 3H).

N-((6-Chloropyridin-3-yl)methyl)-2,6-dimethylimidazo[1,2-*a*]pyridine-3-carboxamide (**4**). To a mixture of (6-chloropyridin-3-yl)methanamine (94 mg, 0.66 mmol) and dichloromethane (20 ml), N,N-diisopropylethylamine (0.246 ml, 1.45 mmol) was added. After stirring for 20 minutes at 0°C, 2,6-dimethylimidazo[1,2-*a*]pyridine-3-carboxylic acid (125 mg, 0.66 mmol), 1-hydroxy-1H-benzotriazol hydrate (151 mg, 0.99 mmol), and DMF (1 ml) were added. The mixture was stirred at 0°C for 1.5 hours and then N-(3-dimethylaminopropyl)-N′-ethylcarbodiimide hydrochloride (189 mg, 0.99 mmol) was added. The reaction was stirred at room temperature for 24 hours. The reaction was quenched and washed with saturated aqueous NaHCO_3_ (2×15 ml), brine (15 ml), and H_2_O (15 ml), dried over MgSO_4_, filtered, and concentrated under reduced pressure. The crude mixture was purified using a silica gel cartridge (Analogix) and eluted with a mixture of methanol/dichloromethane (gradient 0–5%). Collected fractions were evaporated under reduced pressure to obtain the title compound **4** (104 mg, 0.33 mmol, 50% yield) as a white solid. MS (m/z) = 315 ([M+H]^+^); ^1^H NMR: δ 8.87–8.85 (m, 1H), 8.44 (d, 1H, *J* = 2.0) 8.33 (br t, 1H, *J* = 5.8), 7.87 (dd, 1H, *J* = 8.3 and 2.5), 7.52–7.47 (m, 2H), 7.26 (dd, 1H, *J* = 9.1 and 1.5), 4.54 (d, 2H, *J* = 5.8), 2.57 (s, 3H), 2.30 (s, 3H).

### MIC Determination against *Mycobacteria*

The measurement of the minimum inhibitory concentration (MIC) against *M. tuberculosis* strains for each tested compound was performed in 96-well flat-bottom, polystyrene microtiter plates in a final volume of 100 µl. Ten two-fold drug dilutions in neat DMSO starting at 50 mM were performed. Drug solutions were added to Middlebrook 7H9 medium (Difco) and isoniazid (INH) (Sigma Aldrich) was used as a positive control with two-fold dilutions of INH starting at 160 µg/ml. The inoculum was standardized to approximately 1×10^7^ cfu/ml and diluted 1 in 100 in Middlebrook 7H9 broth (Difco). This inoculum (100 µl) was added to the entire plate but G-12 and H-12 wells were used as blank controls. All plates were placed in a sealed box to prevent drying out of the peripheral wells and incubated at 37°C without shaking for six days. A resazurin solution was prepared by dissolving one tablet of resazurin (Resazurin Tablets for Milk Testing; Ref 330884Y′ VWR International Ltd) in 30 ml of sterile PBS (phosphate buffered saline). Of this solution, 25 µl were added to each well. Fluorescence was measured (Spectramax M5 Molecular Devices, Excitation 530 nm, Emission 590 nm) after 48 hours to determine the MIC value.

*M. bovis* BCG strain Pasteur and derivatives were cultured aerobically in Middlebrook 7H9 liquid medium (Difco) containing 0.05% (v/v) Tween-80, 10% (v/v) ADC and 0.25% (v/v) glycerol at 180 rpm and 37°C. To cultivate the *mycobacteria* on solid medium, Middlebrook 7H11 agar (Difco) with 10% (v/v) OADC and 0.5% (v/v) glycerol was used. The MIC of the compounds on solid media was evaluated by plating 10^4^, 10^3^, 10^2^ and 10^1^ bacteria on agar containing different concentrations of compound in a dose response format. The MIC was defined as the lowest concentration of compound resulting in the complete inhibition of bacterial growth. Where applicable, liquid and solid media were supplemented with 25 µg/ml Kanamycin. For all cloning procedures, *E. coli* TOP 10 (Invitrogen) were used exclusively and cultured in LB media containing 50 µg/ml Kanamycin at 180 rpm and 37°C.

### General Antimicrobial Activity Assay

Whole-cell antimicrobial activity was determined by broth microdilution using the Clinical and Laboratory Standards Institute (CLSI) recommended procedure, Document M7-A7, “Methods for Dilution Susceptibility Tests for Bacteria that Grow Aerobically”. Some compounds have been evaluated against a panel of Gram-positive and Gram-negative organisms, including *Enterococcus faecium*, *Enterococcus faecalis*, *Haemophilus influenzae*, *Moraxella catarrhalis*, *Streptococcus pneumoniae*, *Escherichia coli* and *Streptococcus pyogenes*. The MIC was determined as the lowest concentration of compound producing a >80% reduction in growth as observed by OD.

### HepG2 Cytotoxicity Assay

Actively growing HepG2 cells were removed from a T-175 TC flask using 5 ml of Eagle’s MEM (containing 10% FBS/1% NEAA/1% Penicillin+Streptomycin) and dispersed in the medium by repeated pipetting. Seeding density was checked to ensure that new monolayers were not more than 50% confluent at the time of harvesting. The cell suspension was added to 500 ml of the same medium at a final density of 1.2×10^8^ cells per ml. This cell suspension (25 µl, typically 3000 cells per well) was dispensed into the wells of 384-well clear bottom Greiner plates using a Multidrop. Prior to addition of the cell suspension, plates were dispensed with 250 nl of the screening compounds using an Echo 555. Plates were allowed to incubate at 37°C and at a relative humidity of 80% for 48 hours in the presence of 5% CO_2_. After the incubation period, the plates were allowed to equilibrate at room temperature for 30 minutes before proceeding to develop the luminescent signal. The signal developer, CellTiter-Glo™ (Promega) was equilibrated at room temperature for 30 minutes and added to the plates (25 ml per well) using a Multidrop. The plates were left for 10 minutes at room temperature for stabilization and were subsequently read using a ViewLux (Perkin Elmer).

### Microsomal Fraction Stability Experimental Procedure

Pooled mouse, rat, dog and human liver microsomes were purchased from Xenotech. Microsomes (final protein concentration 0.5 mg/ml, MgCl_2_ (final concentration 5 mM) and test compound (final substrate concentration 0.5 mM; final DMSO concentration 0.5%) in 0.1 M phosphate buffer pH 7.4 were pre-incubated at 37°C prior to the addition of NADPH (final concentration 1 mM) to initiate the reaction. The final incubation volume was 600 ml. A control incubation was included for each compound tested where 0.1 M phosphate buffer pH 7.4 was added instead of NADPH (minus NADPH). One control compound was included with each species. All incubations were performed singularly for each test compound. Each compound was incubated for 30 minutes and samples (90 ml) of incubate were taken at 0, 5, 15, 20 and 30 minutes. The control (minus NADPH) was sampled at 30 minutes only. The reactions were stopped by the addition of sample to 200 ml of acetonitrile:methanol (3∶1) containing an internal standard. The terminated samples were centrifuged at 2,500 rpm for 20 minutes at 4°C to precipitate the protein. Quantitative analysis: following protein precipitation, the samples were analyzed using specific LC-MS/MS conditions. Data analysis: from a plot of ln peak area ratio (compound peak area/internal standard peak area) against time, the gradient of the line was determined. Subsequently, half-life and intrinsic clearance were calculated using the equations below:

Elimination rate constant (k) = (− gradient).

Half life (t_1/2_) (min) =  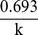


Intrinsic Clearance (CLint) (ml/min/g protein) =  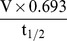 where V = Incubation volume ml/g microsomal protein.

### Pharmacokinetic Studies

For pharmacokinetic studies C57BL/6 female mice of 18–20 g weight were used. Experimental compounds were administered through oral gavage at 50 mg/kg dose at a concentration of 20 ml/kg. All mice received treatment in the fed state. Peripheral total blood was the compartment chosen for the establishment of compound concentrations. Drugs were administered as 1% methyl cellulose suspensions and the blood sampling scheme was: 15, 30 and 45 minutes, 1, 1.5, 2, 3, 4, 8 and 24 hours. At each time-point, blood from 4 different animals was obtained. LC-MS was used as the analytical method of choice for the establishment of compound concentration in blood with a sensitivity of LLQ = 1–5 ng/ml in 25 ml blood. The non-compartmental data analysis was performed with WinNonlin 5.0 and supplementary analysis was performed with GraphPad 4 software. Dose-response studies in an acute murine infection TB model were carried out as previously described [28].

### Generation of *M. bovis* BCG Spontaneous Mutants Resistant to IP 1, 3 and 4

*M. bovis* BCG resistant mutants were generated by plating 10^8^ mid-log (OD_600 nm_, 0.8–1.0) cells on solid media containing 5× MIC of each compound. Resistant colonies were subsequently inoculated into liquid media in the absence of inhibitor. Putative mutants were grown to mid-log and selected on solid media containing 5× MIC of the respective compound to confirm the presence of a mutation conferring resistance to the inhibitor relative to wild-type bacteria. Following the validation of the mutants, the mutant and parental wild-type strains were inoculated in parallel into 50 ml liquid media containing 2× (no inhibitor for wild-type) the MIC of each compound, respectively. Purified genomic DNA from each *M. bovis* BCG spontaneous mutant and the *M. bovis* BCG wild-type (total of seven samples) was prepared for sequencing using the Beckman SPRIworks Fragment Library System I with adapters from the Illumina Multiplexing Sample Preparation Oligonucleotide kit. DNA (3 µg) was fragmented with a Bioruptor instrument (Diagenode, Lie, Belgium) using 100 µl volume and 30 cycles. The fragments were end-repaired, Illumina adapters were ligated and size selected (300–600 bp) using SPRIworks. The size-selected fragments were amplified (18 cycles using Phusion DNA Polymerase) and DNA was purified with Agencourt AMPure XP beads (Beckman Coulter Genomics, High Wycombe, UK). The median fragment size of the final libraries was 420–575 bp (assessed by a BioAnalyzer High Sensitivity LabChip, Agilent). Libraries were quantified with a Quant-iT PicoGreen dsDNA kit (Life Technologies), diluted to 10 nM, pooled and sequenced at the University of Warwick on an Illumina Genome Analyser IIx. Reads were aligned to the *M. bovis* BCG Pasteur 1173P2 reference genome sequence (Genbank accession number NC_008769.1) using the Burrows-Wheeler Aligner (BWA) [29] version 0.5.9rc1 using the aln/sampe pipeline with default settings, other than allowing for insert sizes up to 1000 bases in length. Putative variants were then detected using samtools/bcftools 0.1.17. Aligned BAM files were supplied as input to the samtools mpileup command using the command-line modifiers “-C50 -D -S –E” and otherwise default settings [30]. Low-quality variants were initially filtered to exclude those with a phred-scaled variant quality score of less than 60 and a strand bias score (Fisher’s exact test) of greater than or equal to 0.005. The putative effect of SNPs on coding sequences was determined using snpEff 2.0.5d [31].

### Recombinant DNA Manipulations

Standard molecular techniques were employed during the cloning and DNA manipulations [32]. The production of all oligonucleotide primers and the sequencing of generated constructs was performed by Eurofins MWG Operon; Ebersberg, Germany. *M. bovis* BCG sequence data were obtained from xBASE [33]. For the over-expression of *M. bovis* BCG QcrB in *M. bovis* BCG, three strategies were adopted; *qcrB*, *qcrCAB* and the entire *ctaE-qcrCAB* gene cluster including the predicted promoter region were each cloned into the pMV261 vector (Kanamycin resistance selection marker). The gene and gene clusters were amplified by PCR (Phusion High-Fidelity DNA polymerase; New England Biolabs) from *M. bovis* BCG strain Pasteur genomic DNA using the oligonucleotide primers shown in Table 2. The resulting fragments of sizes 1.7 kb, 3.8 and 4.6 kb were cloned into the pMV261 vector by exploiting the *Bam*HI and *Hind*III restriction sites introduced via the primers (FastDigest restriction endonucleases, Fermentas; T4 DNA ligase, New England Biolabs). All constructs were verified by DNA sequencing. The three constructs produced along with the empty pMV261 vector were electroporated into *M. bovis* BCG as described previously [34].

**Table 2:** Primers used for the construction of pMV261::*qcrB*, pMV261::*qcrCAB* and pMV261::*ctaE-qcrCAB.*

Primer	Sequence (5′→3′)^a^
*qcrB* sense	GATCGATCGGATCCAATGAGTCCGAAACTGAGTCC
*qcrB* anti-sense	GATCGATCAAGCTTCTAGTGCTCGCCGTCTGG
*qcrCAB* sense	GATCGATCGGATCCATTGACGAAACTGGGGTTCACC
*qcrCAB* anti-sense	GATCGATCAAGCTTCTAGTGCTCGCCGTCTGG
*ctaE-qcrCAB* sense	GATCGATCGGATCCTGATGGTCCCATGAGGATCAAC
*ctaE-qcrCAB* anti-sense	GATCGATCAAGCTTGTGCTTGATTTGCCCGGTTC

a Restriction sites used for cloning are underlined (Bam*HI,* Hind*III*).

## Results

### Identification of Imidazo[1,2-*a*]pyridines (IP) as Anti-TB Agents and Optimization

**Figure 2:**
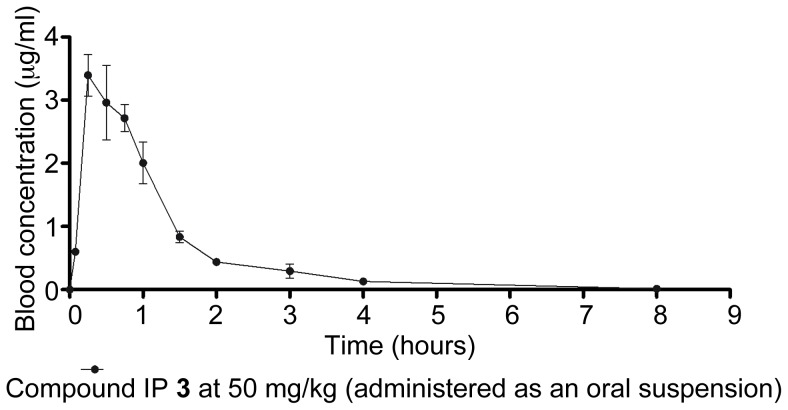
Whole blood pharmacokinetic profile and main parameters of IP 3 after oral administration of a 50 mg/kg suspension in 1% aqueous methylcellulose. Main pharmacokinetic parameters were established after non-compartmental analysis: Cmax (maximum concentration observed in whole blood), 3.4 µg/ml; AUC (Area Under the Curve) (0–8 hours), 4.36 µg.h/ml; F (percentage bioavailability), 61%.

As a result of the recently completed HTS campaign against *M. bovis* BCG with hit confirmation in *M. tuberculosis* H37Rv, a number of chemical families with potential anti-tubercular value were recognized (results to be published elsewhere). Amongst those families, we and others have identified imidazo[1,2-*a*]pyridines (IP) as simple chemical scaffolds amenable to further lead optimization (Figure 1) [10], [23], [24], [25], [26]. A preliminary profiling of the initial IP **1** and IP **2** (Table 1) showed how the compounds were potent and selective anti-tuberculars with no evident signs of cytotoxicity. Despite this interesting profile, the low stability observed in mouse microsomal fractions precluded their progression to the available *in vivo* acute murine TB efficacy model. Medicinal chemistry efforts (details to be reported elsewhere) were hence dedicated to the quick identification of an optimized compound that was able to retain anti-tubercular potency while simultaneously improving the metabolic degradation profile. These efforts led to the identification of IP **3** and **4** (Figure 1, Table 1). The improved *in vitro* metabolic stability found was then confirmed in a murine pharmacokinetic model (Figure 2).

**Figure 3:**
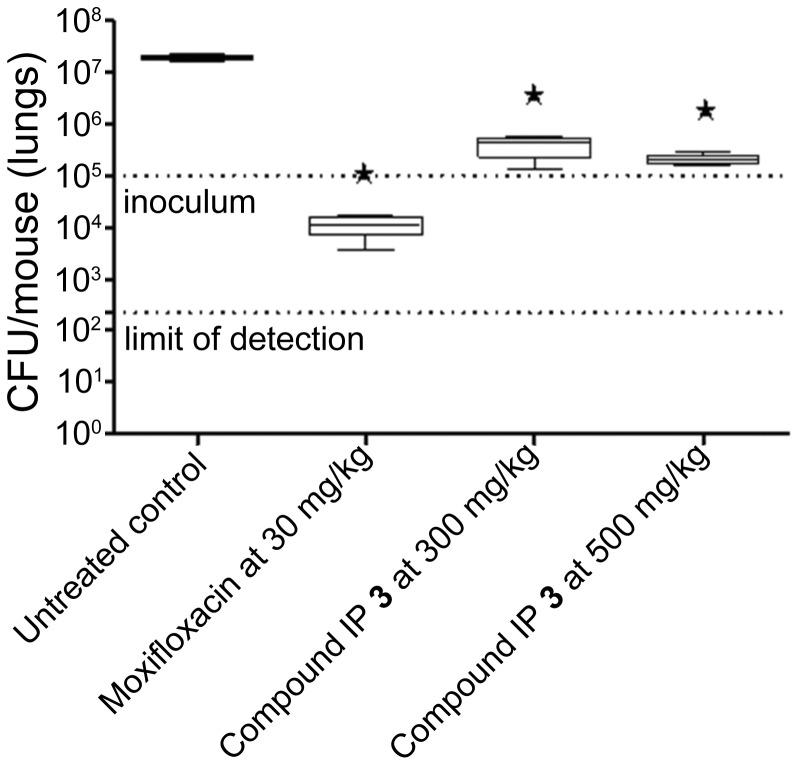
Therapeutic efficacy of anti-tubercular IP 3 against *M. tuberculosis* H37Rv *in vivo*. B6 mice were infected by intra-tracheal instillation with 10^5^ cfu *M. tuberculosis* H37Rv per mouse. The mice were treated with the anti-tubercular orally once a day from day 1 to day 8. Efficacy results are expressed as log cfu reduction in the lungs of infected mice and are the mean±SD of five mice. The shaded line indicates the levels of administered mycobacterial inoculum.

With these parameters in mind, lead compound IP **3** was then progressed to a dose response efficacy study in an acute murine model of TB infection (Figure 3). In this model, IP **3** was shown to have bacteriostatic behavior *in vivo*, demonstrating a 2 log cfu reduction with respect to a non-treated controls both at 300 and 500 mg/kg. No signs of toxicity were observed at any of the administered doses.

### Target Identification of the IP Chemical Family

**Figure 4:**
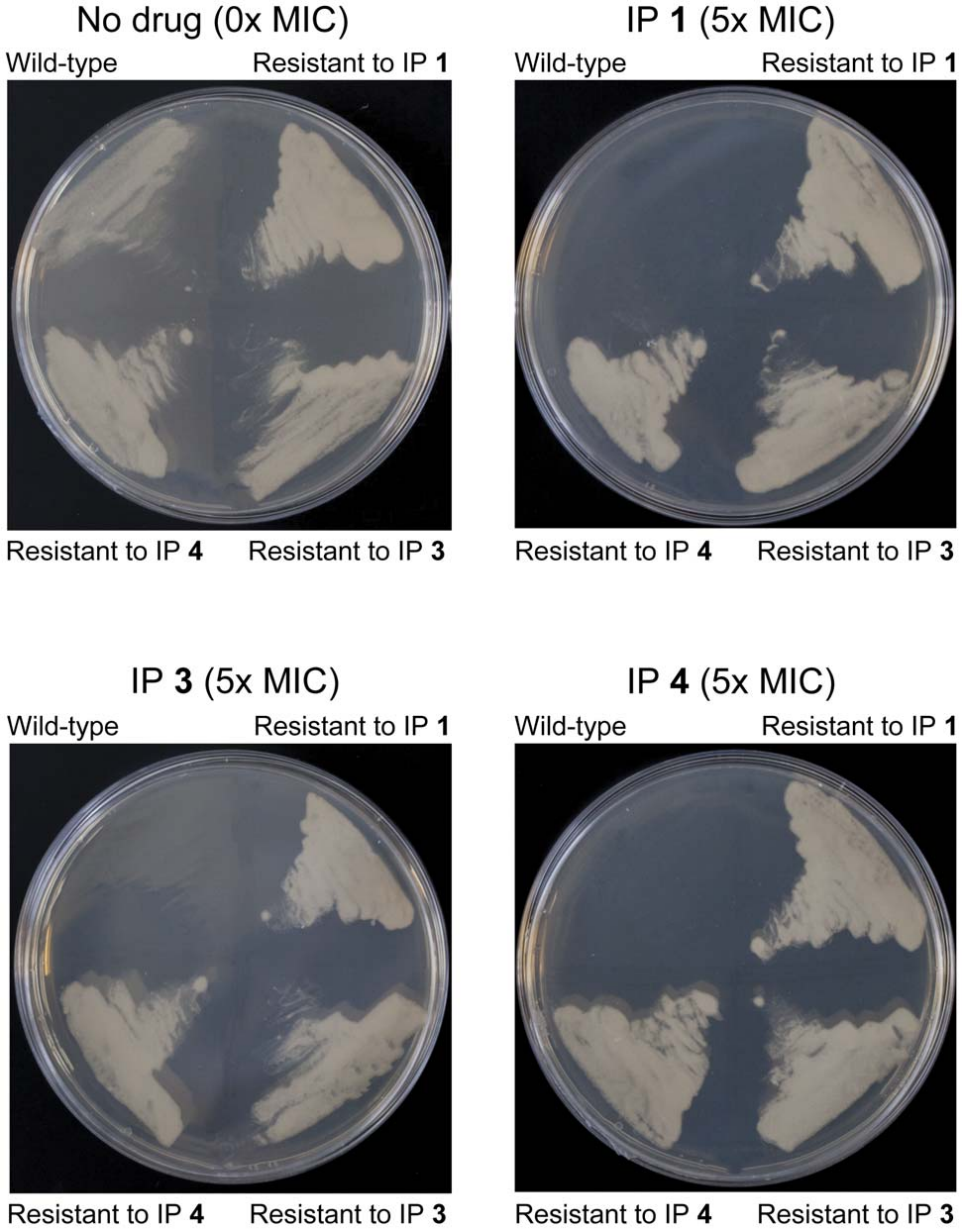
Cross-resistance of *M. bovis* BCG mutants resistant to IP compounds. *M. bovis* BCG resistant mutants raised against IP **1**, **3** and **4** were plated at 5× MIC of each of the respective compounds. A wild-type control demonstrates the resistant nature of the mutants.

The elucidation of the IP target is an important step towards the validation of the inhibitor family as a suitable therapeutic anti-tubercular agent. The three compounds IP **1**, **3** and **4**, exhibited encouraging MIC values against *M. tuberculosis* (Table 1) and thus were selected for use in strategies towards the identification of their target. To establish *M. bovis* BCG as a suitable model for target identification in this study, the MIC of the compounds were determined as stated in Table 1. Using these values, *M. bovis* BCG spontaneous resistant mutants were generated at 5× MIC of each compound. Resistant mutants derived against IP **1**, **3**, and **4** were generated with a frequency of two, three and one respectively in every 1×10^8^ cells. The resistant mutants contained one or more mutations allowing them to survive in the presence of the inhibitor, by either affecting drug accumulation within the cell or by preventing the inhibitory impact of the drug on the target itself. Identification of the mutation locus within the gene could indicate the drug target. Whole-genome sequencing analysis detected 16 putative high-quality single nucleotide polymorphisms (SNPs) compared to the *M. bovis* BCG reference sequence, of which five were likely to have arisen during laboratory storage of the wild-type, parental strain and thus cannot be conferring the phenotype. Three SNPs were predicted synonymous mutations, and two were for putative insertion/deletion events. Of the remaining six putative non-synonymous SNPs, only one was present in all six IP resistant mutants (Table 3). This SNP predicts a single base change in *qcrB* (^937^ACC >^937^GCC) conferring a predicted amino acid alteration (T313A) in the protein sequence. According to TraSH analysis, *qcrB* is an essential gene [35], [36], [37]. Interestingly the mutant allele in this gene was present as a mixture with the wild-type allele in the sequenced population. The stochastic appearance of the other non-synonymous SNPs makes it unlikely that they confer the phenotype and the gene in which they occur can be assumed not the target of the IP family of inhibitors. To confirm QcrB as the target of the three compounds and to establish cross-resistance, the *M. bovis* BCG mutants resistant to IP **1**, **3** and **4** were plated at 5× MIC of each of the respective compounds (Figure 4). The results clearly demonstrate that each *M. bovis* BCG resistant mutant raised against its respective IP was viable in the presence of the other IP family members. This result corroborates that QcrB is the target of the IP family of inhibitors analyzed.

**Table 3:** Single nucleotide polymorphisms detected in *M. bovis* BCG spontaneous mutants resistant to IP compounds.

*M. bovis* BCG chromosome^a^	Codonchange	Amino acid change	Gene	IP 1		IP 3			IP 4
				mutant 1	mutant 2	mutant 1	mutant 2	mutant 3	mutant 1
225198	Gcc/Acc	A103T	*fadD5*	–	–	–	A/T	–	–
663498	Tcc/Ccc	S79P	BCG_0584	–	–	–	S/P	–	–
2439144	Acc/Gcc	T313A	*qcrB*	T/A	T/A	A	T/A	T/A	T/A
3100829	gCc/gTc	A344V	*infB*	A/V	A/V	V	–	–	–
3278815	Ctg/Gtg	L351V	*lipN*	L/V	L/V	V	–	–	–
3856853	gGc/gAc	G294D	BCG_3519	G/D	G/D	D	–	–	–

Wild-type alleles are denoted by a ‘−’ character. Where mixtures of alleles were seen in the population this is indicated, e.g. A/V demonstrates both the genotype coding for alanine and the genotype coding for valine is seen.

a Genomic positions are relative to *M. bovis* BCG str. Pasteur 1173P2 chromosome (Genbank accession: NC_008769.1).

### Over-expression of QcrB in *M. bovis* BCG

**Figure 5:**
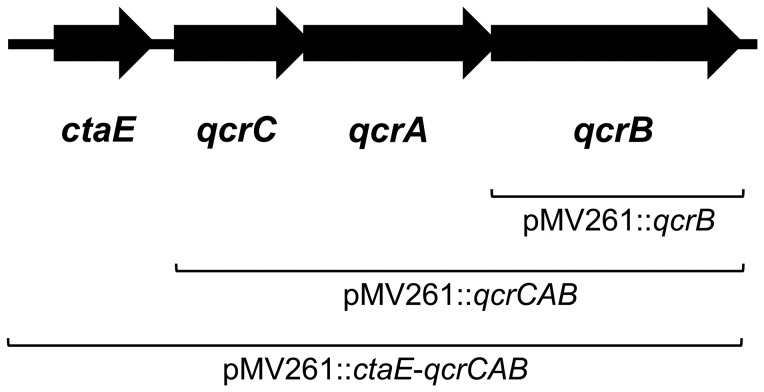
*M. bovis* BCG genome region harboring the genes for the cytochrome *bc*_1_ complex and *ctaE*. The DNA regions cloned into the pMV261 vector are indicated.

**Figure 6:**
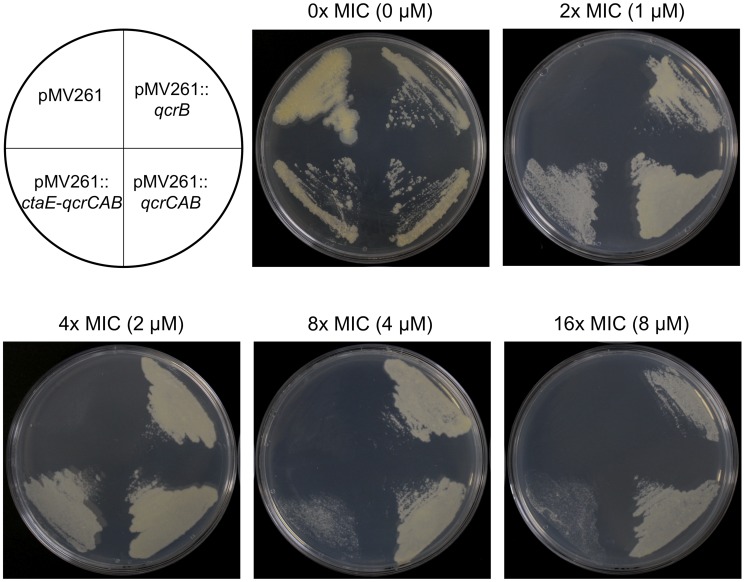
Effect on the MIC of IP 3 during the over-expression of QcrB in *M. bovis* BCG. The over-expression constructs pMV261, pMV261::*qcrB*, pMV261::*qcrCAB* and pMV261::*ctaE-qcrCAB* were electroporated into *M. bovis* BCG and the MIC of IP **3** was evaluated. The plate lay-out is shown (*M. bovis* BCG containing the construct detailed) and the MIC of IP **3** with reference to wild-type *M. bovis* BCG is stated along with the corresponding concentration analyzed. Kanamycin was present at 25 µg/ml to select for the pMV261 vector.

To further support the evidence that QcrB is the target of the IP inhibitor family, QcrB was over-expressed in *M. bovis* BCG using the expression plasmid pMV261 and the MIC of compound IP **3** was reassessed. This inhibitor was selected due to the low quantities of drug required for full anti-tubercular activity (Table 1). QcrB is a putative ubiquinol cytochrome c reductase (subunit b), an integral member of the *bc*_1_ complex of the respiratory electron transport chain. Following studies by Niebisch and Bott [38], it has been reported that in the absence of a functional QcrC, the expression levels of QcrB are severely reduced, implying that QcrC is required in equimolar amounts for the stable assembly of QcrB. As *qcrB* and *qcrC* are members of the same operon, three cloning strategies were adopted to ensure the over-expression of QcrB in a native conformation (Figure 5). The first, *qcrB* alone was cloned into pMV261 (pMV261::*qcrB*). The second, a fraction of the operon encompassing *qcrC*, *qcrA* and *qcrB* (*qcrCAB*) was cloned (pMV261::*qcrCAB*), providing the probable requirement of QcrC for the appropriate integration of QcrB into the membrane. The third, the whole operon, including the putative promoter region of *ctaE,* was cloned into pMV261 vector (pMV261::*ctaE*-*qcrCAB*) to provide the natural promotors of all enzymes, should the vector-encoded promotor region be insufficient to drive protein expression. All constructs were electroporated into *M. bovis* BCG and the MIC of compound IP **3** was assessed to examine the impact of the IP target over-expression (Figure 6). In the absence of QcrB over-expression (pMV261 empty vector control), the MIC of IP **3** was unchanged with that of wild-type *M. bovis* BCG. However, in conditions where the varying constructs of *qcrB* were present in *M. bovis* BCG, the MIC of the respective compound was increased from 0.5 µM to >8 µM (Figure 6). These results further substantiate that QcrB is the target of the IP drug series. Thus, QcrB over-expression has provided supplementary inhibitor target, enabling the bacteria to survive at drug concentrations that would normally be lethal.

## Discussion

The IP compounds described here have been found to display potent anti-tubercular activity against both *M. bovis* BCG and *M. tuberculosis.* Furthermore the target of these inhibitors has been identified as the *b* subunit of the cytochrome *bc*_1_ complex encoded by the gene *qcrB*. The cytochrome *bc*_1_ complex or complex III of the electron transport chain is an integral membrane protein that forms a key component in the bacterial respiratory system. The complex functions as a ubiquinol-cytochrome C reductase, utilizing a catalytic core of three highly conserved components namely the cytochrome c_1_, cytochrome b and the Rieske iron sulfur protein [39]. A Q-cycle mechanism couples electron transfer to proton translocation, adding to the proton electrochemical gradient that is used to generate adenosine triphosphate (ATP). The mechanism of electron transfer within the cytochrome *bc*_1_ complex has previously been described [40], [41].

There are a number of well-characterized inhibitors of the *bc*_1_ complex. The elucidation of their mechanism and crystallographic data identifying binding sites has lead to the development of compounds that inhibit the function of the cytochrome *bc*_1_ complex for therapeutic purposes. These inhibitors tend to target the two catalytic domains utilizing an analogous structure to either quinone or quinol. von Jagow and colleagues [42] first characterized the most widely understood inhibitor of the cytochrome *bc*_1_ complex, myxothiazol, an antibiotic from *Myxococcus fulvus*. Myxothiazol was found to inhibit the oxidant-induced reduction of *b* cytochromes by competitively displacing quinone from the Rieske iron sulfur protein at the high affinity binding site Q_o_ with a K_d_ <1×10^−9 ^M [42]. Many other inhibitors have been identified that function by the same mechanism as myxothiazol, such as mucidin [43], [44], [45], [46] and strobilurin A [44], [47], [48]. Another inhibitor, antimycin [49], has been shown to inhibit the cytochrome *bc*_1_ complex at a different location to myxothiazol, as it functions by binding to the Q_i_ site, proximal to the B_H_ heme, inhibiting oxidation of the cytochrome b subunit [50].

Here we have reported QcrB as the target of the IP family of compounds of the cytochrome *bc*_1_ complex, which was identified by whole genome sequencing of resistant mutants. Resistant mutants raised against all three compounds IP **1**, IP **3** and IP **4** carried an SNP in the *qcrB* gene where a single base change (^937^ACC >^937^GCC) translated to the substitution of a threonine at position 313 for an alanine (T313A) in the cytochrome b subunit (QcrB). *In silico* mapping of this amino acid substitution utilizing the structure of a myxothiazol-bound cytochrome *bc*_1_ complex [51] found the substitution did not fall within the myxothiazol binding site, and therefore resistance is likely conferred by a conformational change as opposed to a mechanistic alteration. SNPs were also identified in other genes. However, due to their inconsistent locations in the genomes of the IP resistant mutants generated and their reported non-essentiality [35], [36], these SNPs were assumed to be non-consequential and not investigated further.

In order to investigate the mechanistic similarities of the three IP compounds in the series, cross-resistance of the genetically dissimilar mutants was established. It was found that all mutants generated were resistant to each of the IP compounds (Figure 4), confirming T313A as the common factor in the resistant phenotype and suggesting all three IP inhibitors function identically. Despite the high potency of the inhibitors, there may be a requirement for future re-engineering and lead optimization now the QcrB target has been identified in this study. Nevertheless, the highly efficient bacterial clearance and novelty of the target as a major component of the electron transport chain shows considerable promise for IP compounds in the treatment of both active and latent phase mycobacterial infection. The latter has been shown to be particularly susceptible to inhibitors of the electron transport chain [52].

Further evidence to support and validate our findings came from the over-expression study of QcrB in *M. bovis* BCG using the mycobacterial vector pMV261, which approximately exerts a 5 times copy number. Three varying length inserts were selected for this study so as to ensure the synthesis of native QcrB, as depicted in Figure 5. *M. bovis* BCG containing an empty pMV261 vector exhibited no change in tolerance to IP **3** in comparison to the wild-type strain, with an MIC of 0.5 µM. On the contrary, there was a marked increase in MIC to >8 µM for *M. bovis* BCG transformants containing all three inserts encompassing *qcrB*. At 16× MIC, we found ample growth from all three inserts, including pMV261::*qcrB*, despite the reported finding that QcrB was only expressed to 10% efficiency if lacking functional QcrC in over-expression vectors [38]. This result clearly confirms QcrB as the target of IP **3** and the other IP compounds: increased expression levels of the IP target enable higher concentrations of IP to enter the cell and bind QcrB without a fatal impact on cell survival.

This study has revealed the IP inhibitor family as a promising anti-tubercular agent, targeting an essential component of the electron transport chain, QcrB. Due to the nature of the target, it is conceivable that the IP compounds could target both active and latent phases of TB infection, an important requirement of future anti-tubercular agents.
